# Unveiling Diverse Trajectories of Internet Addiction and the Influence of Family Environment and Obsessive Beliefs: Multi-Wave Longitudinal Study With Growth Mixed Model

**DOI:** 10.2196/70552

**Published:** 2025-07-23

**Authors:** Chen Tan, Weidan Pu, Qingqian Mo, Xiang Wang, Yonghui Xiang, Shuting Chen, Can Xu, Yichi Zhang, Jinqiang Zhang

**Affiliations:** 1 Department of Clinical Psychology, The Third Xiangya Hospital, Central South University, No. 138 Tongzipo Road, Yuelu District, Changsha, Hunan, 410013, China, 0731-88618696; 2 Medical Psychological Center, Second Xiangya Hospital of Central South University, Changsha, China

**Keywords:** internet addiction, addictive behaviors, predictors, trajectories, longitudinal study, Chinese college students

## Abstract

**Background:**

Recent longitudinal studies have revealed the heterogeneity of the developmental trajectory of internet addiction (IA), which is believed to be due to the influences of interindividual variables. In a social-cognitive framework, family environment (FE) and obsessive beliefs (OBs) are associated with IA severity. However, it remains unclear how these environmental and individual cognition factors interact to influence IA development.

**Objective:**

This study aimed to identify the growth trajectories of IA among college students, considering individual differences over time, and explore how FE and OBs contribute to the identified trajectories.

**Methods:**

A convenience sample of 3575 first-year college students (female: 65.29% [n=2334], mean age 18.7 [SD 0.9]) was recruited, with longitudinal data collected over 3 waves (2019‐2021) and retention rates of 72.4% (n=2585) at T1 and 61.34% (n=2193) at T2. IA trajectories were classified using the latent growth mixture model, and the effects of FE and OBs on the IA intercept and slope were examined by the latent growth curve model. Multivariate logistic regression assessed the predictive effects of FE and OBs on trajectory classification, controlling for sex, residence, and parents’ education. Furthermore, structural equation modeling was used to map the road from FE and OBs to follow-up IA.

**Results:**

Latent growth mixture model uncovered 4 distinct trajectories: high-risk (5.09%), medium to high-risk (29.85%), medium to low-risk (35.95%), and low-risk (29.11%), while latent growth curve model revealed that both FE and OBs significantly influenced IA initial level (intercept: *β*_FE_cohesion/ conflict_=−0.169/−0.191, *P*<.001; *β*_OBs_responsibility/ control of thoughts_=0.129/0.279, *P*<.05) and development rate (slope: *β*_FE_conflict_=0.073, *P*<.05; *β*_OBs_ control of thoughts_=−0.165, *P*<.001). Furthermore, logistic regression showed that compared with the low-risk group: high-risk students exhibited reduced cohesion (odds ratio [OR] 0.831, 95% CI 0.721-0.957; *P*<.01), elevated conflict (OR 0.866, 95% CI 0.745-1.006; *P*<.05), and lower independence (OR 0.841, 95% CI 0.710-0.996; *P*<.05); medium-high risk showed higher conflict (OR 0.890, 95% CI 0.826-0.959; *P*<.01) and OBs (OR_responsibility_ 1.020, 95% CI 1.003-1.037; OR_control of thoughts_ 1.028, 95% CI 1.010-1.045; *P*<.01); and medium-low risk had increased conflict (OR 0.911, 95% CI 0.841-0.986; *P*<.05). Moreover, structural equation modeling demonstrated a significant partial mediation effect of OBs on the relationship between FE and follow-up IA (effect _T0/ T1/ T2_=−0.03/−0.02/ −0.02, *P*<.001).

**Conclusions:**

This study reveals 4 heterogeneous IA trajectories among college students, influenced by both FE and OBs through their effects on the IA initial level and development rate. Notably, FE not only influences IA development directly but also exerts its influence indirectly through the mediation of OBs. These findings highlight the necessity of targeted interventions addressing family environmental risk factors and maladaptive OBs in youth for IA.

## Introduction

Internet addiction (IA) is defined as a pattern of uncontrollable or excessive engagement with the internet that results in subjective distress or interference in major areas of life functioning [1]. Youth in Chinese colleges have high internet accessibility and frequently spend substantial time actively engaging in web-based activities, leading to a high prevalence rate of IA ranging from 7.2% to 21.16% in this cohort [2-6]. IA is associated with various adverse outcomes in youth, including depression [7-9], poor sleep quality [10,11], and academic functioning [12], and a decline in self-esteem and self-confidence [13]. Studies suggest that IA evolves as a dynamic process rather than remaining a static behavioral pattern [14]. Longitudinal studies on youth during the college stage are essential for exploring the developmental trajectory and influencing factors of IA, which enables the establishment of predictive models, offering a scientific foundation for precise intervention on IA.

Different studies hold varying perspectives on the developmental trends of IA among college students. Traditional variable-centered analyses have estimated the general developmental trajectory of IA [15,16], such as a U-shape developmental trajectory. However, such average trends may obscure the heterogeneity of individual development, meaning not all adolescents follow the same trajectory. Understanding this heterogeneity is crucial for both theory and practice, as distinct subgroups may require tailored prevention and intervention strategies. Recent person-centered approaches have revealed the heterogeneity of IA developmental trajectories [17,18]. By applying latent class growth modeling (LCGM) or latent growth mixture modeling (LGMM), studies have identified 2 to 6 distinct trajectories of IA [12,17-22]. For example, a 2-year prospective investigation among Chinese college students identified 3 trajectories of IA: low-level, moderate-level, and high-level score trajectories [21]. Another long-term study of Singaporean adolescents reported 6 latent subgroups, 3 of which were in pathological status for at least a year [22]. These findings raise two critical scientific questions: (1) Which interindividual variables influence IA developmental trajectories and their underlying heterogeneity? (2) How do these variables affect IA trajectories (eg, initial levels, development rates, and classification)?

Ecological systems theory posits that adolescent behavior is shaped by layered environmental systems, with the family as the primary microsystem [23]. Family environment (FE), as a crucial factor affecting individual development, has been proven to have a substantial and enduring influence on IA [24]. Cross-sectional data have shown that a negative FE characterized by low levels of family satisfaction and cohesion and a high level of conflict between parents and children is significantly associated with IA amongst adolescents [25-29]. Prior research suggests that adolescent addictions tend to increase in families marked by weak emotional bonds (ie, low cohesion) in response to situational and developmental stress [30]. Recent longitudinal studies provide further support for the predictive effect of familial factors on the IA development and its trajectory classification [12,31,32]. For instance, Geng et al [12] demonstrated that parental conflict was a negative familial predictor of IA risk trajectories (eg, “moderate-increasing” and “high-increasing” groups). A 3-year longitudinal study of Hong Kong adolescents found that positive family functioning predicted lower probabilities of IA [32]. Adolescents from families with low cohesion, high conflict, or emotional neglect may show higher initial IA levels as they seek emotional support or escape on the internet [2,33]. If these patterns persist, they can also accelerate IA growth over time, especially under stressful life events or interpersonal problems [34]. However, it remains elusive how the FE influences IA trajectory development, including the IA development rate starting from its initial level in the youths during the college stage. In this study, we aimed to address this gap by not only exploring the influence of FE on IA trajectory classification but also examining its impact on the initial level and development rate. This more delicate examination helps to confirm the role of family factors in the developmental process of IA, providing a scientific basis for more personalized interventions for adolescents dealing with IA.

Beyond environmental factors, personal cognitive strategy is also regarded as a cardinal role involved in IA development [35]. Previous studies have identified various cognitive factors related to IA, including low impulse control ability [36], cognitive attentional bias [36], high tendency for risky decision-making [36,37], and poor cognitive emotion regulation ability [38]. However, these cognitive characteristics are commonly involved in a wide range of mental health issues such as depression and anxiety and therefore may lack specificity in explaining the mechanisms underlying IA. In contrast, obsessive beliefs (OBs) represent a more specific cognitive style that may play a critical role in IA. These beliefs include inflated responsibility, overestimation of threat, excessive control of thoughts, and perfectionism. They have been recognized as cognitive vulnerability factors in the onset and maintenance of obsessive-compulsive disorder (OCD) [39-44]. Importantly, compulsivity, which refers to repetitive behaviors performed with reduced voluntary control, is a core feature shared by both OCD and behavioral addictions [45]. Furthermore, recent neuroimaging studies have revealed a functional reorganization of the cortico-striato-thalamo-cortical circuitry in individuals with addiction, characterized primarily by an abnormal transition from the ventral striatum to the dorsal striatum [46-48]. This neural transition is considered a core neuropathological mechanism underlying the progression of addictive behaviors from an occasional behavior to a compulsive pattern, highlighting the central role of compulsive cognitive processing of addiction-related cues in the development of addictive behaviors [46,47]. Although the association between OCD symptoms and IA has been explored in prior studies [49-52], the specific role of OBs in the development of IA has not been systematically examined. Therefore, this study aims to investigate how OBs influence both the occurrence and progression of IA. This research seeks to clarify the unique contribution of OBs in IA development and provide new directions for cognitive-based intervention strategies.

Social cognitive theory (Bandura) emphasizes the triadic interaction between environment, cognition, and behavior [53]. Furthermore, the family is the primary place for individuals’ socialization and plays a critical role in the development of cognitive style [54-56]. A large body of research has shown a significant relationship between negative family factors and maladaptive cognitive styles [57-59]. For example, individuals from families with conflict-indifferent relationships report more negative automatic thoughts than those from harmonious families [60]. A 21-day daily diary study reports that negative self-relevant cognition such as self-blame increases with elevation of interparental conflict in adolescents [61]. Although previous studies have not directly examined the relationship between FEs and OBs, the robust evidence linking family factors to various cognitive styles supports the assumption that negative FEs may contribute to the development of OBs. In this study, guided by ecological systems theory and social-cognitive models, we further hypothesize the mediating role of OBs in the relationship between FE and longitudinal IA development.

In this study, we longitudinally tracked a sample of 3575 college students over a 2-year period across 3 time points. The primary objective is (1) to examine the heterogeneity in the developmental trajectories of IA among college students over time; (2) to elucidate the effects of FE and OBs on the developmental trajectory of IA, with a specific focus on the initial level, development rate, and classification predictions; and (3) to explore the mediating role that OBs might play in the relationship between FE and follow-up IA. We hypothesized the following.

- H1: Significant heterogeneity in IA developmental trajectories would be observed in college students, with distinct subgroups showing different patterns of change over time.
- H2: FE and OBs would significantly influence the initial levels and development rates of IA over time.
- H3: FE and OBs would significantly predict IA trajectory classification, even after controlling for sociodemographic variables.
- H4: OBs would exert a mediating role between FE and longitudinal IA (T0, T1, and T2).

## Methods

### Participants

This study adopted a longitudinal design with first-year students from a university in Hunan, selected through convenience sampling. The data collection protocol comprised 3 waves: baseline assessment in October 2019 (T0), first follow-up in December 2020 (T1), and second follow-up in December 2021 (T2). Initially, 3575 freshmen participated at T0 (female: 65.29% [n=2334], male: 34.71% [n=1241]). Subsequent assessments revealed an attrition rate of 17% at T1 (2967 retained) and 12% at T2 (3146 retained). The final analysis included 2193 participants (61.34% of the original cohort) who completed assessments at all 3 waves.

### Ethical Considerations

All procedures adhered to the ethical guidelines outlined in the Declaration of Helsinki and were approved by the Ethics Committee of the Second Xiangya Hospital of Central South University (approval number 81671341). Following the acquisition of informed consent, the questionnaires were administered in classrooms under the supervision of trained psychology postgraduate students. Data collection was conducted anonymously to ensure participants’ privacy and confidentiality. Participation was entirely voluntary, and participants were informed that they could withdraw from the study at any time without penalty. Counseling support was made available to participants.

### Measures

#### Internet Addiction Test (T0, T1, and T2)

IA was assessed using the 20-item internet addiction test [62]. The scale comprises 20 items, and responses were rated on a 5-point Likert scale (1 = “never,” 5 = “always”). Participants who obtained a score of less than 30 points indicated nonrisk of IA. Scores between 31 and 49 points were considered as mild IA, and scores between 50 and 79 were moderate IA. The scores between 80 and 100 indicate a severe IA. In the previous literature, the internet addiction test demonstrated adequate reliability and validity in both adults and youths [63,64]. In this study, the scale demonstrated good reliability, with Cronbach α of 0.909, 0.933, and 0.938 for T0, T1, and T2, respectively.

#### Family Environment Scale (T0)

The 27-item Chinese version of the Family Environment Scale was employed to assess the FE, covering 3 dimensions: cohesion, independence, and conflict. The second-level scoring scale (0‐1) was used. For the conflict dimension, reverse coding was applied (ie, values of 0 were recoded to 1, and values of 1 to 0), so that higher scores indicate lower levels of family conflict. As a result, higher total scores across all dimensions reflect a more positive FE. High reliability and factorial validity of the Family Environment Scale have been reported in Chinese adolescent students [65]. In this study, the Cronbach α was 0.757 at T0.

#### Obsessive Beliefs Questionnaire-44 (OBQ-44) (T0)

The obsessive beliefs questionnaire-44 gauges dysfunctional beliefs linked to the origin and persistence of obsessions and compulsions. This self-report instrument comprises 3 subscales: responsibility/ threat estimation (RT), perfectionism/ certainty, and importance/ control of thoughts (ICT). The respondents are requested to score themselves the degree to which the situation described in each statement on a 7-point scale (1=disagree very much; 7=agree very much). With scores ranging from 44 to 308, higher values indicate stronger compulsive beliefs. Obsessive beliefs questionnaire-44 has demonstrated good psychometric properties in the general population [66]. In this study, the Cronbach α was 0.924 at T0.

### Data Analyses

We calculated the means, SDs, and correlations between main variables across 3 time points in SPSS (version 25.0; IBM). *P* values were adjusted using the false discovery rate correction with a significance threshold of *P*<.05 to control for multiple comparisons. According to the aims of this study, we conducted the following analyses in Mplus (version 8.3; Muthén and Muthén) [67]. First, IA trajectory classification was analyzed by LGMM. Second, the effects of FE and OBs on the initial level and development rate of IA trajectory were examined by the latent growth curve model (LGCM) [68]. Maximum Likelihood estimation with robust standard errors was employed to handle nonnormally distributed data and estimate model parameters, with missing data addressed via Full Information Maximum Likelihood in the LGMM and LGCM analyses [67]. The model fit indices and analysis process are detailed in the Supplemental Materials.

Furthermore, the Multivariate Logistic Regression Model was applied using the R3-step to examine the predictive effect of FE and OBs on trajectory classification after controlling for gender, residence, and parents’ education level [69]. Finally, structural equation modeling was used to explore the predictive effect of FE and OBs on the IA (T0, T1, and T2, respectively). A multicollinearity test showed that there was no evidence of multicollinearity among the independent variables. Sociodemographic characteristics were incorporated as control variables in the model. The mediation effects were validated with 5000 bias-corrected bootstrap samples (CI excluding 0 denoted significance).

## Results

### Descriptive Statistics

The mean age of all participants at baseline was 18.00 years (SD 0.74), with 71.96% female. Attrition analyses indicated that missing data were random (Little’s MCAR test: *P*=.16) [70], and no substantial demographic differences were found between retained and dropped-out participants, except for gender and father’s education. Demographic characteristics are detailed in Table S1 in Multimedia Appendix 1. Across all 3 waves, IA was positively correlated with OBs and negatively correlated with FE, and other correlations are presented in Table S2 in Multimedia Appendix 1.

### The Heterogeneous Trajectories of IA

In this study, LGMMs with 1‐6 latent classes were separately estimated, and the summary of fit indices is presented in Table S3 in Multimedia Appendix 1. From the 1-class to the 4-class model, the information indices Akaike information criterion, Bayesian information criterion, and sample-size adjusted Bayesian information criterion gradually decreased, while the Bayesian information criterion value started to increase in the 5-class and 6-class models. Additionally, we found a significant bootstrap-based likelihood ratio test in 2-class to 4-class, while the Lo-Mendel-Rubin likelihood ratio test results suggested that the 2-class and 4-class solutions were suitable. Further observation confirmed that the entropy value (almost 0.80) was the highest when retaining the 4-class model. By comparing multiple indices as described above and comprehensively considering the parsimony, accuracy, and practical significance of the models, this study ultimately selected the 4-class classification model as the optimal one.

**Figure 1.**
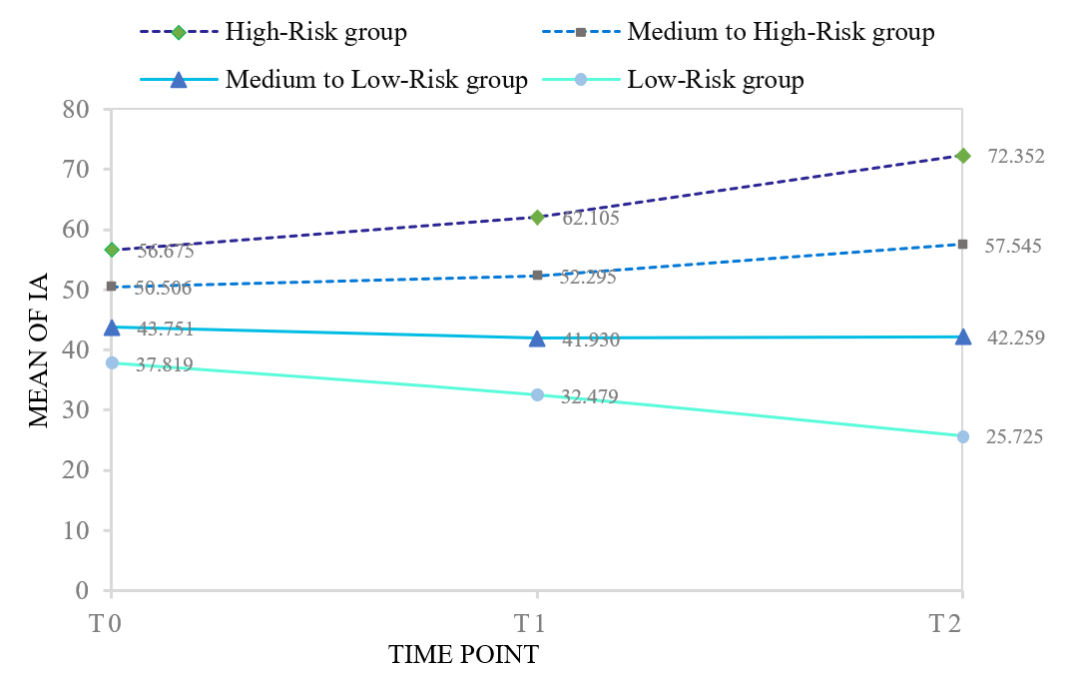
Trajectories of IA in each latent category over 3 time points. IA: internet addiction.

Figure 1 shows the estimated growth parameters and trajectories for each of the 4 classes, and Table S4 in Multimedia Appendix 1 presents the intercept and slope for each latent trajectory category, respectively. The first latent category, termed high-risk group (5.09% of the sample, n=112), maintained a high level of IA and exhibited an upward trend over time (*M*_intercept_ = 56.09, *P*<.001; *M*_slope_ = 7.97, *P*<.001); The second, labeled medium to high-risk group (29.85% of the sample, n=655), sustained a medium to high level of IA and showed a slow upward trend (*M*_intercept_ = 50.08, *P*<.001; *M*_slope_ =3.62, *P*<.001); The third, termed medium to low-risk group (35.95% of the sample, n=788), maintained a moderate to low level of IA and exhibited a slow downward trend (*M*_intercept_ =43.49, *P*<.001; *M*_slope_ =−0.68, *P*=.01); The fourth, termed low-risk group (29.11% of the sample, n=638), sustained a relatively low level of IA and displayed a downward trend (*M*_intercept_ =37.99, *P*<.001; *M*_slope_ =−6.09, *P*<.001).

### Family Environment and Obsessive Beliefs on IA Trajectory

**Figure 2.**
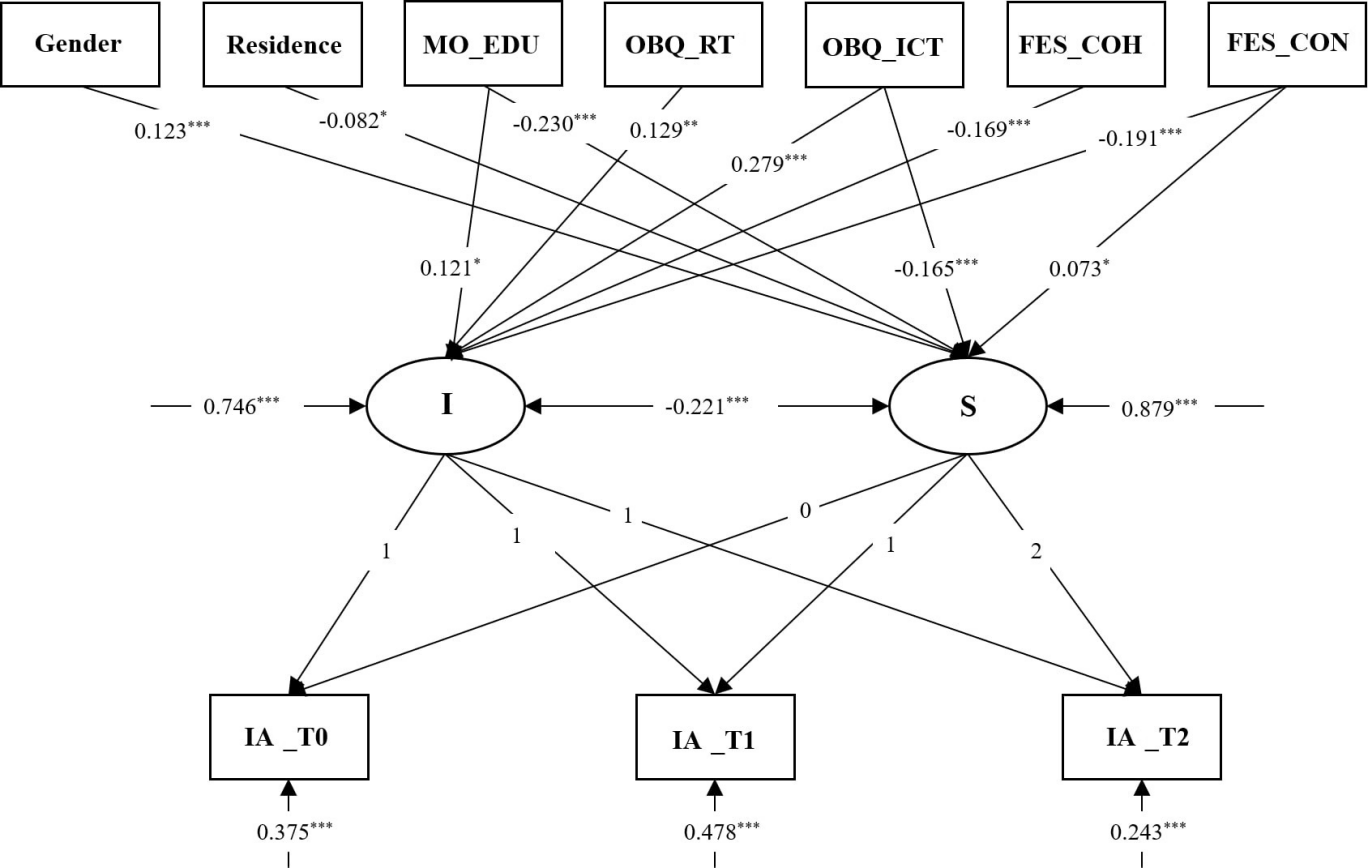
Conditional latent growth curve model of IA. The figure only shows significant paths. FES_COH: family environment scale_ cohesion; FES_CON: family environment scale_ conflict (FES_CON was reverse-coded prior to analysis. Higher scores indicate less family conflict, ie, better family environment. Odds ratios reflect this reverse scoring). I: intercept; IA: internet addiction; MO_EDU: mother’s education level; OBQ_ICT: obsessive beliefs questionnaire_ importance/ control of thoughts; OBQ_RT: obsessive beliefs questionnaire_ responsibility/ threat estimation; S: slope. *P<.05, **P<.01, ***P<.001.

Based on the correlation analysis results, this study integrated sociodemographic characteristics (such as gender, residence, and parents’ education level), FE factors, and OBs as covariates into the LGCM. A conditional model, illustrated in Figure 2, was developed to assess how these covariates predict both the initial levels and development rates of IA among college students. The conditional model exhibited a good fit, *χ*^2^_11_=60.087, comparative fit index=0.976, Tucker-Lewis index=0.928, root mean square error of approximation=0.045, standardized root mean square residual=0.021.

OBs, along with FE characteristics and the mother’s education level, were significant predictors of the initial IA score. Specifically, higher levels of mother’s education (*β*_intercept_=0.121, *P*<.05), OBs (RT and ICT) (*β*_intercept_=0.129, *P*<.01; *β*_intercept_=0.279, *P*<.001) and poor FE (cohesion, conflict) (*β*_intercept_=−0.169 *P*<.001; *β*_intercept_ = −0.191, *P*<.001) were associated with higher initial IA score.

Furthermore, gender (*β*_slope_=.123, *P*<.001), long-term residence (rural vs urban) (*β*_slope_=−0.082, *P*<.05), mother’s education level (*β*_slope_=−0.230, *P*<.001), ICT (*β*_slope_=−0.165, *P*<.001) and family conflict (*β*_slope_=0.073, *P*<.05) significantly predicted the growth rate of IA. This indicates that IA development changes more rapidly for females than males, and for individuals from rural rather than urban areas. Additionally, lower mother’s education level, ICT, and family conflict were associated with a more rapid change in the IA trajectory.

### Family Environment and Obsessive Beliefs on Distinct IA Trajectories

Tables1^3^ present the results of multivariate logistic regression analysis. Compared with the low-risk group, college students in the high-risk group (regression coefficient [*B*]=0.885, *P*<.01), medium to high-risk group (*B*=0.550, *P*<.001; odds ratio [OR] 1.734, *P*<.01) and medium to low-risk group (*B*=0.687, *P*<.001; OR 1.988, 95% CI 1.486-2.659 ; *P*<.001) were more likely to be female. College students in the medium to high-risk group were more likely to live in the rural areas than in the urban areas (*B*=–0.269, *P*<.05; OR 0.764, 95% CI 0.597-0.977; *P*<.05). The high-risk group reported poorer FE, characterized by lower cohesion (*B*=–0.186, *P*=.01; OR 0.831, 95% CI 0.721-0.957; *P*<.01), higher conflict (OR 0.866, 95% CI 0.745-1.006, *P*<.05), and reduced independence (*B*=−0.173, *P*<.05; OR 0.841, 95% CI 0.710-0.996; *P*<.05). The medium to high-risk group had higher family conflict (*B*=−0.117, *P*<.01; OR 0.890, 95% CI 0.826-0.959; *P*=.001), as well as elevated RT (*B*=0.020, *P*<.05; OR 1.020, 95% CI 1.003-1.037; *P*<.05) and ICT (*B*=0.027, *P*<.01; OR 1.028, 95% CI 1.010-1.045; *P*<.01). Students in the medium to low-risk group experienced higher family conflict (*B*=−0.094, *P*<.05; OR 0.911, 95% CI 0.841-0.986; *P*<.05) compared with the low-risk group.

**Table 1.** Multiple logistic regression results for the high-risk group using the low-risk group as a reference.

	*B^a^*	95% CI	OR^b^ (95% CI)
Gender	0.885^c^	0.232 to 1.538	2.423 (1.261 to 4.656)
Residence	–0.400	–1.010 to 0.211	0.671 (0.364 to 1.235)
FA_EDU^d^	–0.120	–0.401 to 0.161	0.887 (0.670 to 1.175)
MO_EDU^e^	–0.233	–0.546 to 0.101	0.800 (0.579 to 1.106)
FES_COH^f^	–0.186^c^	–0.327 to –0.044	0.831^c^ (0.721 to 0.957)
FES_CON^g^	–0.144	–0.294 to 0.006	0.866^i^ (0.745 to 1.006)
FES_IND^h^	–0.173^i^	–0.343 to –0.004	0.841^i^ (0.710 to 0.996)
OBQ_RT^j^	0.005	–0.032 to 0.043	1.005 (0.968 to 1.044)
OBQ_PC^k^	0.032	–0.006 to 0.071	1.033 (0.994 to 1.073)
OBQ_ICT^l^	0.021	–0.011 to 0.053	1.021 (0.989 to 1.054)

a B: Unstandardized regression coefficient.

b OR: odds ratio.

c *P*<.01.

d FA_EDU: father’s education level.

e MO_EDU: mother’s education level.

f FES_COH: family environment scale_ cohesion.

g FES_CON: family environment scale_ conflict (FE_CON was reverse-coded prior to analysis. Higher scores indicate less family conflict (ie, better family environment). Odds ratios reflect this reverse scoring.

h FES_IND: family environment scale_ independence.

i *P*<.05.

j OBQ_RT: obsessive beliefs questionnaire_ responsibility/threat.

k OBQ_PC: obsessive beliefs questionnaire_ perfectionism/certainty.

l OBQ_ICT: obsessive beliefs questionnaire_importance/control of thoughts.

**Table 2.** Multiple logistic regression results for the medium to high-risk group using the low-risk group as a reference.

Variable	*B* ^a^	95% CI	OR^b^ (95% CI)
Gender	0.550^c^	0.278 to 0.823	1.734^d^ (1.320 to 2.277)
Residence	–0.269^e^	–0.515 to –0.023	0.764^e^ (0.597 to 0.977)
FA_EDU^f^	0.032	–0.092 to 0.156	1.033 (0.912 to 1.169)
MO_EDU^g^	–0.052	–0.156 to 0.051	0.949 (0.855 to 1.053)
FES_COH^h^	–0.059	–0.140 to 0.022	0.943 (0.870 to 1.023)
FES_CON^i^	–0.117^d^	–0.191 to –0.042	0.890^c^ (0.826 to 0.959)
FES_IND^j^	–0.033	–0.108 to 0.042	0.967 (0.897 to 1.043)
OBQ_RT^k^	0.020^e^	0.003 to 0.036	1.020^e^ (1.003 to 1.037)
OBQ_PC^l^	–0.013	–0.029 to 0.002	0.987 (0.972 to 1.002)
OBQ_ICT^m^	0.027^d^	0.010 to 0.044	1.028^d^ (1.010 to 1.045)

a B: regression coefficient.

b OR: odds ratio.

c *P*<.001.

d *P*<.01.

e *P*<.05.

f FA_EDU: father’s education level.

g MO_EDU: mother’s education level.

h FES_COH: family environment scale_ cohesion.

i FES_CON: family environment scale_ conflict (FE_CON was reverse-coded prior to analysis. Higher scores indicate less family conflict (ie, better family environment). Odds ratios reflect this reverse scoring.

j FES_IND: family environment scale_ independence.

k OBQ_RT: obsessive beliefs questionnaire_ responsibility/threat.

l OBQ_PC: obsessive beliefs questionnaire_ perfectionism/certainty.

m OBQ_ICT: obsessive beliefs questionnaire_importance/control of thoughts.

**Table 3.** Multiple logistic regression results for the medium to low-risk group using the low-risk group as a reference.

Variable	*B* ^a^	95% CI	OR^b^ (95% CI)
Gender	0.687^c^	0.396 to 0.978	1.988^c^ (1.486 to 2.659)
Residence	–0.102	–0.294 to 0.089	0.903 (0.745 to 1.093)
FA_EDU^d^	–0.076	–0.195 to 0.043	0.927 (0.823 to 1.044)
MO_EDU^e^	–0.059	–0.149 to 0.031	0.943 (0.862 to 1.031)
FES_COH^f^	–0.014	–0.105 to 0.077	0.986 (0.900 to 1.080)
FES_CON^g^	–0.094^h^	–0.174 to –0.014	0.911^h^ (0.841 to 0.986)
FES_IND^i^	–0.011	–0.085 to 0.063	0.989 (0.919 to 1.065)
OBQ_RT^j^	0.004	–0.013 to 0.021	1.004 (0.987 to 1.022)
OBQ_PC^k^	0.007	–0.009 to 0.022	1.007 (0.991 to 1.022)
OBQ_ICT^l^	0.004	–0.013 to 0.021	1.004 (0.988 to 1.021)

a B: regression coefficient.

b OR: odds ratio.

c *P*<.001.

d FA_EDU: father’s education level.

e MO_EDU: mother’s education level.

f FES_COH: family environment scale_ cohesion.

g FES_CON: family environment scale_ conflict (FE_CON was reverse-coded prior to analysis. Higher scores indicate less family conflict (ie, better family environment). Odds ratios reflect this reverse scoring.

h *P*<.05.

i FES_IND: family environment scale_ independence.

j OBQ_RT: obsessive beliefs questionnaire_ responsibility/threat.

k OBQ_PC: obsessive beliefs questionnaire_ perfectionism/certainty.

l OBQ_ICT: obsessive beliefs questionnaire_importance/control of thoughts.

### The Mediating Effect of Obsessive Beliefs Between Family Environment and IA

**Figure 3.**
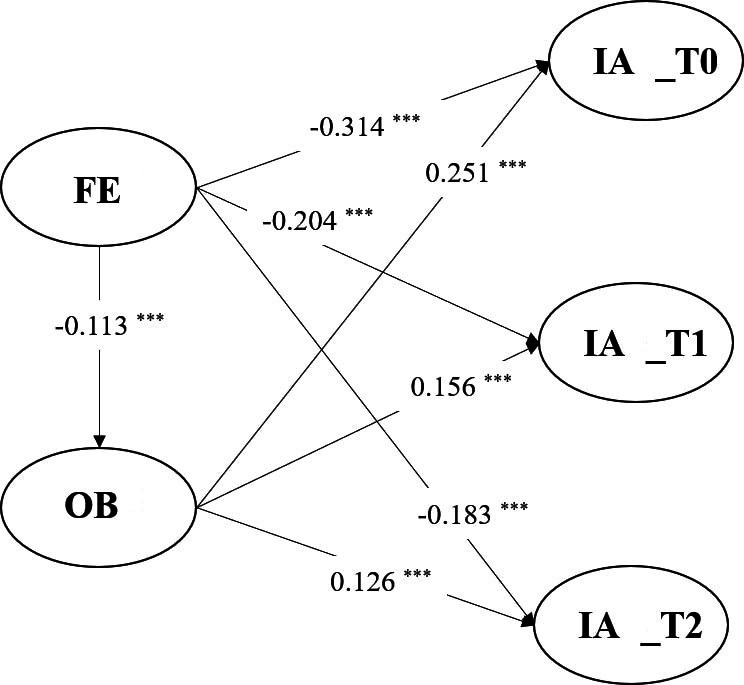
Structural equation modeling of the relationship between FES, OBQ, and followed-up IA. The figure above shows the standardized path coefficients for significant paths, with gender, residence, and parents’ education level included as control variables. FE: family environment; IA: internet addiction; OB: obsessive beliefs. ***P<.001.

As shown in Figure 3, the FE had a significant negative effect on both OBs (*β*=−0.113, *P*<.001) and IA at 3 times of survey (T0, T1, and T2) (*β*=−0.314; −.204; −.183, *P*<.001). While OBs positively predicted IA at 3 times of survey (*β*=0.251; .156; .126, *P*<.001). The bootstrap results with 5000 resamples revealed that FE exerted a significant indirect effect on IA in 3 waves (T0, T1, and T2), due to the mediating effect of OBs (effect_T0_=−0.03, 95% CI −0.46 to −0.16; effect _T1_ = −0.02, 95% CI −0.35 to −0.12; effect _T2_ =−0.02, 95% CI −0.33 to −0.10). Thus, OBs partially mediated the relationship between FE and IA. The model fitting indexes and path coefficients are shown in Table S5 and Table S6 in Multimedia Appendix 1, respectively.

## Discussion

### Principal Findings

This study reveals a predominant declining trend of IA growth trajectory among college students, with 4 distinct categories emerging. Importantly, our study demonstrates that both FE and OBs significantly affect the initial level and development rates of IA developmental trajectory, further delineating the process of how environment and cognition influence IA development. Finally, consistent with our hypothesis, we show that OBs exhibit a significant mediating effect on the influence of FE on IA. The partial mediating model indicates that the FE not only influences the IA development directly but also modifies the individual cognitive style (ie, OB), which finally facilitates the IA progression indirectly.

### The Trajectories of IA

Consistent with previous findings, first-year college students demonstrated relatively high baseline levels of IA [71], likely due to increased autonomy and greater access to the internet after graduating from high school. However, a gradual decline in IA severity was observed over time, which may reflect developmental transitions such as increased maturity, enhanced self-regulation, and adaptation to university life. This pattern aligns with prior research suggesting that the risk of IA typically rises during early adolescence but tends to decline in late adolescence or early adulthood as individuals gain better emotional and behavioral control. Additionally, the significant negative correlation between IA’s initial level and development rate suggests that individuals with higher baseline IA tend to show slower increases or decreases over time, while those with lower initial IA are more likely to escalate. This may reflect a ceiling effect or spontaneous remission in high-risk individuals and highlights the importance of monitoring low-level users who may develop IA later.

This study identified 4 distinct developmental trajectories of IA, which align with findings from Hong et al [72] and Zhou et al [73]. In contrast, some researchers have identified 3 subgroups among adolescents—low-decreasing, moderate-increasing, and high-increasing trajectories [12]—while others have reported only 2 IA trajectories (ie, low/decreasing and high/decreasing) [19]. Variations in the number and nature of identified trajectories across studies may be attributed to several factors, including differences in sample sociodemographic characteristics (eg, age, cultural contexts), personality and cognitive styles, as well as measurement instruments, assessment intervals, and the number of time points. These methodological and contextual differences may lead to the identification of either more nuanced or more general trajectory classifications. Understanding these developmental patterns is essential for designing targeted interventions and support systems that address the diverse needs of individuals at varying levels of IA risk.

In our sample, approximately 5.09% of students followed a high-risk trajectory of IA, characterized by an initially severe level of symptoms followed by a rapid escalation over time. This proportion closely aligns with the national prevalence of severe IA (approximately 6.3%) [74]. A similar high-risk trajectory has been observed in previous studies [12,75], lending further support to the existence of a small yet highly vulnerable subgroup. These individuals are likely to experience significant impairments in psychological well-being, academic performance, and social functioning. The steep and worsening trajectory underscores the urgent need for early identification and targeted interventions to prevent further deterioration and chronicity of IA.

In contrast, the medium to high-risk group (29.85%) exhibited a moderate level of IA symptoms at baseline that increased slightly over time, maintaining a state of “mild to moderate” addiction. Similar developmental patterns have been reported in prior studies [12,17,22,72,73]. This trajectory may reflect students who, under the influence of academic pressure, interpersonal difficulties, or emotional distress, gradually adopt more entrenched patterns of problematic internet use. Although not meeting clinical thresholds for IA, the upward trend indicates potential risk for future deterioration. Regular screening and early-stage psychosocial interventions—such as those embedded within school mental health services—are recommended to prevent escalation.

The medium to low-risk group (35.95%) demonstrated a moderate initial severity of IA that declined slightly over time, maintaining an overall “mild addiction” status. This trajectory suggests a degree of self-regulation and recovery. Comparable patterns have been reported in earlier work [22,72,76], suggesting that some students may possess strong internal coping capacities or benefit from external protective factors—such as peer support or successful academic adaptation—that contribute to behavioral improvement. While these students are not at immediate high risk, they may reside at the “risk margin,” warranting continued attention through psychoeducation, digital literacy programs, and developmental support to consolidate positive behavioral changes.

Finally, the low-risk group (29.11%) followed a decreasing trajectory, beginning with a low level of IA and trending toward remission. This pattern reflects a trajectory of psychological resilience and healthy development. Consistent with earlier findings [12,19,20,72], such individuals are typically classified as “stable-healthy.” The resilience demonstrated by this group may be attributed to various protective factors—such as effective emotion regulation, supportive family dynamics, or limited exposure to web-based risk environments—and warrants further exploration to inform universal prevention strategies.

### Sociodemographic Characteristics on IA Trajectories

We explored the influence of several sociodemographic characteristics, including gender, residence, and parents’ education level, on IA trajectory, although this is not the focus of this study. For gender, no significant differences in baseline IA levels were found between males and females. However, over the 2-year follow-up period, IA symptoms developed more rapidly among females compared with males, and females were more likely to fall into the high-risk IA trajectory group. This finding is consistent with previous studies [77-79], suggesting that females may be at greater risk for developing IA than males. Such differences may stem from gender-specific patterns of web-based activity and coping strategies. For instance, females tend to use the internet more frequently for maintaining social connections, making them more susceptible to emotionally driven triggers, whereas males are more likely to engage in gaming and competitive web-based activities [80]. Further, youths from rural areas exhibited a faster development of IA compared with those who grew up in urban areas and were more likely to fall into the medium-to-high-risk categories. The growing penetration rate of the internet, along with a large population of left-behind children in rural areas in China [81], may make the IA increasingly prominent in children and adolescents [82,83]. Moreover, we also showed that higher maternal education was associated with higher initial IA levels and slower progression of IA, suggesting that mothers with high education levels may provide earlier chances to bring their children into the internet world, but play a protective role against IA in the development of youths ultimately.

### Family Environment on IA Trajectories

Consistent with the family system theory [84], our findings highlight FE as a critical determinant in the development of IA. Most notably, the observation of predictive effects of FE on IA initial level and development rate in this study highlights a potential mechanism in which FE may exert a persistent influence throughout the entire IA developmental process in youth. Thus, this study underscores the necessity of early and sustained attention to the FE as a cornerstone of intervention and prevention strategies. Subgroup analysis identifies protective factors such as cohesion and independence within the family, aligning with previous research [85] and underscoring the adaptive role of a positive FE in IA development [86]. Conversely, family conflict significantly elevates the risk of IA. Longitudinal studies further emphasize that individuals experiencing pronounced conflicts in parent-child relationships during early adolescence are more prone to developing IA symptoms in mid-adolescence [87]. A possible explanation is that adolescents may use excessive internet use as a coping mechanism for family strife, transforming into a maladaptive strategy over time [88].

### Obsessive Beliefs on IA Trajectories

Based on the relationship between obsessive-compulsive symptoms and IA [50], this study pioneers an exploration into the direct impact of OBs on the development of IA. We found that OBs significantly shape both the initial levels and development rates in IA, highlighting the critical need for early and ongoing attention to these cognitive patterns. Upon subgroup analysis, students with elevated control thinking were more likely to fall into “high-risk” or “medium-high risk” categories compared with their low-risk counterparts. The significance of these cognitive patterns underscores the notion that individuals bear responsibility for their ideas and their ensuing consequences. Excessive control thinking during web-based activities may impede individuals from discontinuing their behavior on the web, leading to addiction. Furthermore, college students expressing heightened levels of responsibility and threat assessment are prone to the “medium-to-high risk” group. This inflationary responsibility aligns with Salkovskis’ cognitive theory, positing that the evaluation of intrusive thoughts leads individuals to assume responsibility for the outcomes of such thoughts and perceived danger. Inflated responsibility, in turn, triggers automatic negative thoughts and discomfort [89,90]. The burden of excessive responsibility in the real world, coupled with the allure of the perceived simplicity and safety of the web-based realm, may propel individuals toward increased participation in the internet, thereby facilitating the emergence and progression of IA.

However, no significant relationship of IA development was observed with perfectionism in our data. Yang et al [91] identified a direct relationship between maladaptive perfectionism and IA among Chinese college students. A plausible explanation for this inconsistency could be the nuanced nature of perfectionism. Perfectionism encompasses various dimensions, such as self-oriented perfectionism, other-oriented perfectionism, and socially prescribed perfectionism, each exerting different influences on behaviors and attitudes [92]. In the specific context of IA development, it is conceivable that particular dimensions of perfectionism might have a more pronounced impact than others. For example, self-oriented perfectionism, characterized by setting high personal standards, may foster a conscientious approach to web-based activities without necessarily leading to addictive behaviors. Conversely, socially prescribed perfectionism, driven by perceived external expectations, might create pressure and stress, potentially fueling the development of IA. Further research could dissect specific aspects of perfectionism within the context of IA, examining their interplay with other cognitive elements for a nuanced comprehension of their contributions to the trajectories of IA.

### Meditation of Obsessive Beliefs Between Family Environment and IA

In accordance with the social-cognitive model [93], the mediation analysis in this study confirms that FE, as a social background, can indirectly influence IA through the cognitive pathway of OBs. The findings reveal that OBs mediate the relationship between FE and IA at all 3 time points, indicating that FE persistently exerts a direct impact on college students’ IA, as well as an indirect effect by modifying cognitive patterns such as OBs.

This evidence suggests the importance of multisystemic efforts (ie, addressing social context and intrapersonal cognitive factors simultaneously) to prevent or reduce adolescents’ IA. Intervention strategies targeting IA among college students could include cognitive therapy to reduce OBs or family therapy to mitigate familial conflicts, thereby interrupting the developmental trajectory of IA.

### Strengths and Implications

This study offers 3 key contributions to the understanding of IA development. First, by employing LGMM, we identified 4 distinct IA trajectories (high-risk, medium to high-risk, medium to low-risk, and low-risk), revealing the heterogeneity in how IA unfolds among college students. Second, building on prior research linking OCD symptoms and IA, this study is among the first to systematically demonstrate the independent predictive role and mediating function of OBs in IA progression. Our findings highlight how cognitive vulnerabilities—such as inflated responsibility and excessive need for control—can exacerbate compulsive internet use and drive the longitudinal escalation of IA. Third, by integrating ecological systems theory with the social-cognitive model, we propose a dual-pathway mechanism: FE influences IA both directly—affecting the initial level and development rate—and indirectly, through its impact on OBs. This framework bridges the gap between macro-level contextual influences and micro-level cognitive processes, offering a more integrative etiological account of IA. Finally, the findings provide actionable insights for prevention and intervention. They underscore the necessity of tailoring strategies to specific risk profiles. For instance, individuals with highly OBs or those exposed to family conflict may benefit from cognitive-behavioral interventions targeting maladaptive cognitions, combined with family-based approaches that enhance cohesion and reduce conflict. Furthermore, the protective effect of maternal education suggests that empowering parents—especially mothers—through digital literacy and parenting programs could serve as a valuable preventive resource.

### Limitations and Further Research

This study has several limitations. First, it was conducted in a single university in Hunan Province, which may limit the generalizability of the findings. Regional and cultural factors in China (such as rural-urban disparities in digital access and educational pressure) may influence students’ internet use patterns and IA risk [94,95]. For example, students from rural areas may have had less prior exposure to technology, while academic stress in Chinese universities may promote excessive internet use as a coping strategy. Future studies should include more diverse samples across different regions and cultural contexts. Second, although gender has been included as a covariate in the analysis, our findings showed a significant predictive effect of gender on the IA trajectory, suggesting the potential influence of gender imbalance (approximately 72% female) on the IA trajectory cannot be fully ruled out in this study. It calls for future studies with gender-balanced samples to clarify this issue more elegantly. Third, this study did not assess comorbid mental health issues such as depression and anxiety [17,96]. Future research could investigate how IA codevelops with other internalizing (eg, depressive symptoms) and externalizing (eg, substance use) disorders using multi-trajectory frameworks and explore bidirectional relationships between IA and comorbid symptoms to identify central nodes (ie, cognition distortions, sleep disturbances) as intervention targets. Fourth, due to the limited number of time points (3 waves), we were unable to estimate more complex developmental trajectories such as quadratic or nonlinear models. Future longitudinal studies with more frequent assessments are encouraged to address this limitation.

### Conclusions

This study offers crucial insights into the development of adolescent IA, highlighting that IA is not a “one-size-fits-all” issue. It underscores the vital function of a supportive FE in mitigating IA risks among youth, while also revealing the detrimental impact of maladaptive cognitive patterns, like OBs, on the onset and development of IA. Our findings provide empirical evidence to help clinicians identify high-risk individuals by monitoring vulnerability factors, including elevated OBs, a lack of emotional warmth in the family, exposure to family conflict, and persistently high IA symptoms across follow-up assessments. Tailored interventions such as cognitive-behavioral therapy targeting maladaptive beliefs and family-based programs aimed at enhancing family cohesion may be effective for at-risk groups. Taken together, this research advances theoretical models of IA by integrating ecological and cognitive perspectives and offers practical implications for developing more individualized and effective prevention and intervention strategies for youth.

## Supplementary material

10.2196/70552 Multimedia Appendix 1 Supplementary materials.

## Abbreviations

B: regression coefficient
FE: family environment
IA: internet addiction
ICT: importance/ control of thoughts
LGCM: latent growth curve model
LGMM: latent growth mixture model
OB: obsessive belief
OCD: obsessive-compulsive disorder
OR: odds ratio
RT: responsibility/ threat estimation
