# Supplementary material for: Unveiling Diverse Trajectories of Internet Addiction and the Influence of Family Environment and Obsessive Beliefs: Multi-Wave Longitudinal Study With Growth Mixed Model
Source: J Med Internet Res. 2025 Jul 23;27:e70552. doi: 10.2196/70552 (PMC12309421; doi:10.2196/70552)
Supplement: Multimedia Appendix 1 [file jmir-v27-e70552-s001.pdf]

Supplemental Materials for

Unveiling diverse trajectories of internet addiction and the influence of family environment and obsessive beliefs: a multi-wave longitudinal study with growth mixed model

Authors' note:

We developed these materials to provide additional technical information and to keep the main manuscript focused.

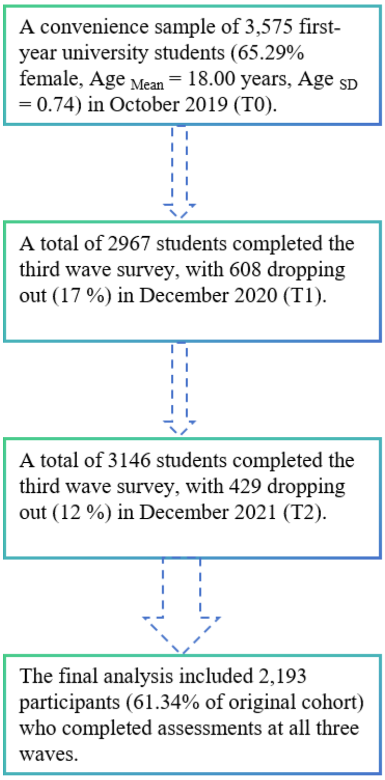

Figure S1 Sampling procedure for the three-wave longitudinal study

Table S1 Sociodemographic characteristics of participants (*N*=2193)

Variables	Frequency	Percentage
**Gender**		
Male	615	28.04%
Female	1578	71.96%
**Long-term place of residence**		
Rural	1216	55.45%
Urban	967	44.09%
Miss	10	0.46%
**Father's education level**		
Uneducated	24	1.09%
Primary school	414	18.88%
Middle school	806	36.75%
High school	473	21.57%
Junior college	239	10.90%
Undergraduate	217	9.90%
Graduate student or above	20	0.91%
**Mother's education level**		
Uneducated	55	2.51%
Primary school	557	25.40%
Middle school	824	37.57%
High school	375	17.10%
Junior college	225	10.26%
Undergraduate	140	6.38%
Graduate student or above	17	0.78%

Table S2 Descriptive statistics and correlational analyses of study variables (*N*=2193)

	1	2	3	4	5	6	7	8	9	10	11	12	13
1 FE_COH	--												
2 FE_CON	0.58 [h]	--											
3 FE_EXP	0.25 [h]	0.25 [h]	--										
4 [a] FE	0.83 [h]	0.84 [h]	0.58 [h]	--									
5 OB_RT	-0.06	-0.11 [h]	-0.00	-0.08 [h]	--								
6 OB_PC	-0.02	-0.10 [h]	-0.01	-0.06 [g]	0.76 [h]	--							
7 OB_ICT	-0.09 [h]	-0.11 [h]	-0.06 [g]	-0.11 [h]	0.68 [h]	0.60 [h]	--						
8 [b] OB	-0.06 [g]	-0.12 [h]	-0.02	-0.09 [h]	0.92 [h]	0.90 [h]	0.84 [h]	--					
9 [c] T0_IA	-0.25 [h]	-0.27 [h]	-0.14 [h]	-0.30 [h]	0.26 [h]	0.20 [h]	0.30 [h]	0.28 [h]	--				
10 [d] T1_IA	-0.17 [h]	-0.18 [h]	-0.06 [g]	-0.19 [h]	0.16 [h]	0.14 [h]	0.20 [h]	0.18 [h]	0.49 [h]	--			
11 [e] T2_IA	-0.15 [h]	-0.16 [h]	-0.07 [h]	-0.18 [h]	0.14 [h]	0.11 [h]	0.15 [h]	0.15 [h]	0.42 [h]	0.58 [h]	--		
12 FA_EDU	0.03	0.03	0.03	0.04	-0.05 [f]	0.01	-0.05 [f]	-0.03	-0.02	-0.06 [g]	-0.05 [g]	--	
13 MO_EDU	0.03	0.03	0.01	0.03	-0.04	0.00	-0.06 [g]	-0.03	-0.03	-0.06 [g]	-0.06 [g]	0.65 [h]	--
M	7.01	6.37	6.05	19.51	60.34	60.35	36.97	157.7	44.72	43.34	43.75	3.55	3.29
SD	2.00	2.13	1.49	4.33	12.99	13.11	10.78	32.82	13.8	14.54	14.96	1.27	1.24

Notes: FE, family environment; FE_COH, family environment_ cohesion; FE_CON, family environment_ conflict; FE_EXP, family environment_ expression/independence; OB, obsessive beliefs; OB_RT, obsessive beliefs_ responsibility/threat estimation; OB_PC, obsessive beliefs_ perfectionism/certainty; OB_ICT, obsessive beliefs_ importance/control of thoughts; IA, internet addiction; FA_EDU, father's education level; MO_EDU, mother's education level; *M*, means; *SD*, standard deviations; [a] FE: 39 participants have missing data, with a total of 46 missing values; [b] OB: 114 participants have missing data, with a total of 174 missing values; [c] T0_IA: 62 participants have missing data, with a total of 70 missing values; [d] T1_IA: 75 participants have missing data, with a total of 79 missing values; [e] T2_IA: 49 participants have missing data, with a total of 54 missing values; [f] $P < .05$; [g] $P < .01$; [h] $P < .001$.

Attrition analyses

Independent-samples t-tests and chi-square tests were conducted to compare baseline sociodemographic characteristics and main study variables between participants who completed all three waves (n = 2,193) and those who dropped out (n = 1,382). The results found that no significant differences were observed in age (t = -1.15, P = .25) and residence (χ^2 = 3.51, P = .061). A significant difference was found in gender (χ^2 = 109.21, P < .001), which may be due to the overall gender imbalance in the sample (approximately 72% female). No significant group differences were found for mother's education (χ^2 = 12.74, P = .121), while a small but statistically significant difference was observed in father's education (χ^2 = 15.21, P = .033). In terms of key psychological variables, independent-samples t-tests showed no significant differences between retained and dropped participants for IA (t = 1.395, P = .163), obsessive beliefs (t = -0.386, P = .700), or family environment (t = -1.944, P = .052).

Latent Growth Mixture Modeling (LGMM)

To investigate the heterogeneous characteristics of the time - varying trends of IA and to test whether there are latent classes of heterogeneity in the overall developmental trajectory of IA among college students, this study further established a LGMM using *Mplus* 8.3. The LGMM encompasses two types of latent variables: continuous latent variables and categorical latent variables. Specifically, the continuous latent variables, manifested as random intercept and random slope factors, are employed to describe the initial differences and development trends. On the other hand, the categorical latent

variables serve to depict the heterogeneity of the population by segregating the population into mutually exclusive latent class subgroups.

The evaluation metrics of the LGMM include information indices and test statistics. The information indices consist of the Akaike Information Criterion (AIC), Bayesian Information Criterion (BIC), Sample - Size Adjusted Bayesian Information Criterion (aBIC), and entropy (Nylund et al., 2007). The values of AIC, BIC, and aBIC are utilized for model comparison, with smaller values indicating better model fit. The entropy value represents the classification accuracy of the model (McDonald and Lochman, 2012). When entropy $\geqslant 0.8$, it indicates that the classification accuracy exceeds 90% (Wu et al., 2022). The test statistics include the Lo - Mendel - Rubin Likelihood Ratio Test (LMR) and the Bootstrap - based Likelihood Ratio Test (BLRT). When the LMR and BLRT values of a model reach a significant level ($p < 0.05$), it implies that model k explains a higher proportion of variance compared to model k - 1, indicating that the k - class model is markedly superior to the k - 1 - class model. Additionally, sample sizes within each class were deemed to be needed to be greater than 5% to be interpretable/generalizable. (Nylund et al., 2007).

Latent Growth Curve Modeling (LGCM)

Longitudinal trajectories of IA were analyzed using LGCM in *Mplus 8.3*. In the LGCM, two latent variables were estimated to model individual developmental trajectories of IA: the intercept and the slope. The intercept represents the initial level of IA symptoms—i.e., an individual's estimated IA score at the baseline assessment (T0). It captures between-person differences at the starting point of the study. The slope

reflects the rate and direction of development (e.g., growth rate) in IA symptoms over time across the three time points (T0, T1, T2). A positive slope indicates an increase in IA symptoms, whereas a negative slope suggests a decrease. To explore predictors of IA trajectories, sociodemographic covariates (gender, residence, parental education level) and key theoretical variables (family environment, obsessive beliefs) were incorporated into a conditional LGCM. Gender (male = 1, female = 2) and parental education (1 = no formal education; 2 = elementary school; 3 = junior high; 4 = senior high; 5 = vocational college; 6 = bachelor's degree; 7 = postgraduate) were dummy-coded.

Model parameters were estimated using Maximum Likelihood estimation with Robust standard errors (*MLR*). The goodness-of-fit indices for LGCM were tested using χ^2/df, comparative fit index (CFI), Tucker Lewis index (TLI), mean square error of approximation (RMSEA), and standardized root mean square residual (SRMR). CFI > 0.90 and TLI > 0.90 indicate acceptable model fit, and that two values >0.95 indicate good model fit. RMSEA and SRMR values should be lower than 0.08 and 0.06, respectively (Hu and Bentler, 1999).

The overall trajectory of IA

The LGCM demonstrated an adequate fit [χ^2 = 10.934 (P < .001), Root Mean Square Error of Approximation (RMSEA) = 0.067, Comparative Fit Index (CFI) = 0.993, Tucker Lewis' index (TLI) = 0.980, Standardized Root Mean Square Residual (SRMR) = 0.015]. As shown in Figure S1, the estimated mean values of intercept (M = 44.463, P < .001) and slope (M = -0.558, P < .001) of LGCM revealed an average initial

IA level of 44.463, with a significant downward trend from T0 to T2. And a significant negative association between the intercept and slope (-15.568, $P < .001$). This suggests that individuals with higher initial levels (intercept) may exhibit slower rates of change (slope) over time, or vice versa. The variance for intercept ($\sigma^2 = 114.654$, $P < .001$) and slope ($\sigma^2 = 27.618$, $P < .001$) underscored notable individual differences in the initial state and change rate of IA over time.

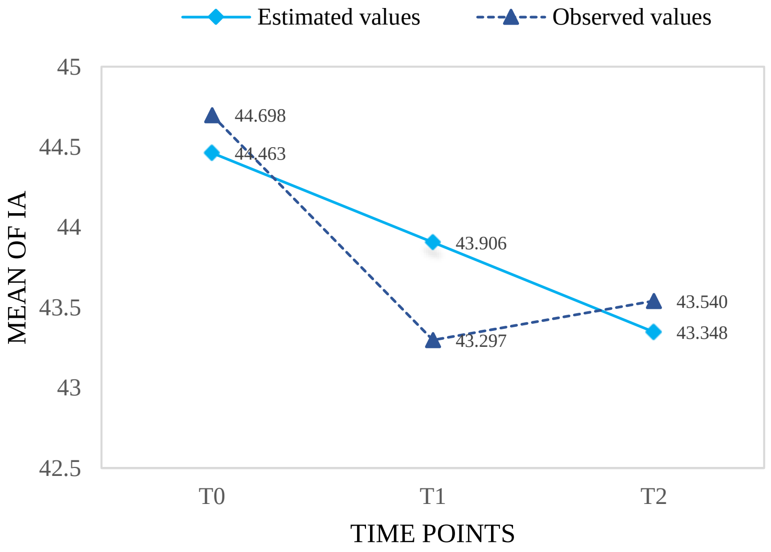

Figure S2 Trajectories of IA over three time points

Table S3 Fitting results of Latent Growth Mixture Model (LGMM)

Category	K	Log(L)	AIC	BIC	aBIC	Entropy	LMR	BLRT	Membership percentages (%)
1C	8	-26139.6	52295.18	52340.72	52315.3	1	—	—	—
2C	11	-26083.8	52189.56	52252.18	52217.23	0.598	0	0	0.477/ 0.523
3C	14	-26067.6	52163.27	52242.97	52198.49	0.617	0.357	0	0.214/ 0.401/ 0.385
**4C**	**17**	**-26026.4**	**52086.79**	**52183.57**	**52129.56**	**0.792**	**0**	**0**	**0.051/ 0.360/ 0.298/ 0.291**
5C	20	-26015.1	52070.23	52184.08	52120.54	0.750	0.019	0	0.239/ 0.051/ 0.213/ 0.247/ 0.250
6C	23	-26010.4	52066.88	52197.81	52124.74	0.645	0.189	0	0.057/ 0.142/ 0.290/ 0.051/ 0.248/ 0.212

Notes: K, the number of freely estimated parameters; Log (L), the logarithmic likelihood value; AIC, Akaike Information Criterion; BIC, Bayesian Information Criterion; aBIC, adjusted Bayesian Information Criterion; LMR, Lo - Mendel - Rubin Likelihood Ratio Test; BLRT, Bootstrapped Likelihood Ratio Test.

Table S4 Intercept and slope of each potential category

Category	Intercept		Slope	
	B	*SE*	*B*	*SE*
High-Risk group	56.09 [b]	2.00	7.97 [b]	1.22
Medium to High-Risk group	50.08 [b]	0.58	3.62 [b]	0.36
Medium to Low-Risk group	43.49 [b]	0.55	-0.68 [a]	0.27
Low-Risk group	37.99 [b]	0.51	-6.09 [b]	0.30

Notes: *B*, Estimate; *SE,* Standard Error; [a] $P < .05$, [b] $P < .001$.

Table S5 Model Fit Indicators of Structural Equation Modeling (SEM)

	χ^2/df	CFI	TLI	RMSEA	SRMR
T0	4.296	0.978	0.966	0.039	0.027
T1	3.363	0.983	0.975	0.033	0.025
T2	3.004	0.986	0.978	0.030	0.024

Notes: χ^2/df, Chi-Square to Degrees of Freedom Ratio; CFI, Comparative Fit Index; TLI, Tucker-Lewis Index; RMSEA, Root Mean Square Error of Approximation; SRMR, Standardized Root Mean Square Residual.

Table S6 Structural Equation Modeling (SEM) analysis results

Paths					Bootstrapping 95% CI		
	B	*β*	*SE*	*Z*	Lower	Upper	*P*
FE → OB	-0.91	-0.11	0.24	-3.86	-1.38	-0.45	< .001
FE → IA_T0	-2.89	-0.31	0.25	-11.57	-3.42	-2.43	< .001
OB → IA_T0	0.29	0.25	0.03	10.82	0.24	0.34	< .001
Mediation of OB between FE and IA_T0	-0.26	-0.03	0.07	-3.75	-0.40	-0.13	< .001
FE →OB	-0.91	-0.11	0.24	-3.74	-1.42	-0.45	< .001
FE → IA_T1	-1.98	-0.20	0.25	-7.80	-2.48	-1.50	< .001
OB → IA_T1	0.19	0.16	0.03	7.03	0.14	0.24	< .001
Mediation of OB between FE and IA_T1	-0.17	-0.02	0.05	-3.43	-0.28	-0.08	< .001
FE → OB	-0.91	-0.11	0.25	-3.73	-1.41	-0.44	< .001
FE → IA_T2	-1.84	-0.18	0.27	-6.89	-2.43	-1.37	< .001
OB → IA_T2	0.16	0.13	0.03	6.01	0.10	0.21	< .001
Mediation of OB between FE and IA_T2	-0.14	-0.01	0.04	-3.36	-0.23	-0.06	< .001

Notes: CI, Confidence Interval; *B*, Unstandardized Coefficient; *β*, Standardized Coefficient; *SE*, Standard Error; *Z*, Estimate/ Standard Error.

We conducted a sensitivity analysis on the conditional Latent Growth Curve Model (conditional LGCM) using the leave - one - out method. We sequentially removed different sociodemographic characteristic variables to observe changes in the model fit indices and model results, and obtained the following findings. Overall, after sequentially removing different sociodemographic characteristic variables, although there were certain changes in the model fit indices and model results, the overall trend remained relatively stable. This indicates that the model is somewhat robust to the removal of these variables. Among them, OBQ_ICT and FES_CON had a significant impact on both the intercept and slope in multiple models, demonstrating the stability of their importance in the model.

Table S7 Model fit indices of conditional LGCM

Model	χ^2/df	CFI	TLI	RMSEA	SRMR	AIC	BIC	aBIC
M1	5.462	0.976	0.928	0.045	0.021	51567.148	51726.412	51637.452
M2	2.644	0.992	0.975	0.028	0.011	50964.129	51111.717	51029.112
M3	6.004	0.975	0.926	0.048	0.021	51572.287	51720.175	51637.570
M4	6.003	0.975	0.926	0.048	0.018	51578.516	51726.404	51643.799
M5	5.830	0.976	0.929	0.047	0.021	51564.424	51712.311	51629.706

Notes: χ^2, Chi-square; *df*, degree of freedom; CFI, Comparative Fit Index; TLI, Tucker-Lewi's index; RMSEA, Root Mean Square Error of Approximation; SRMR, Standardized Root Mean Square Residual; AIC, Akaike Information Criterion; BIC, Bayesian Information Criterion; aBIC, Sample-Size Adjusted BIC; M1: The Conditional LGCM (integrated all sociodemographic characteristics); M2: The Conditional LGCM (leave gender out); M3: The Conditional LGCM (leave residence out); M4: The Conditional LGCM (leave mo_edu out); M5: The Conditional LGCM (leave fa_edu out).

Table S8 Model results of conditional latent growth curve modeling

Model	Effect	I		S	
		β	*SE*	*β*	*SE*
	Gender	0.013	0.024	0.123 [c]	0.030
	Residence	-0.006	0.026	-0.082 [a]	0.033
	FA_EDU	-0.028	0.028	0.032	0.034
M1	MO_EDU	0.121 [a]	0.049	-0.230 [c]	0.060
	OBQ_RT	0.129 [b]	0.041	-0.043	0.051
	OBQ_PC	-0.038	0.038	0.034	0.047
	OBQ_ICT	0.279 [c]	0.034	-0.165 [c]	0.042

	FES_COH	-0.169 ^{c}	0.030	0.069	0.036
	FES_CON	-0.191 ^{c}	0.030	0.073 ^{a}	0.037
	FES_EXP	-0.030	0.047	-0.023	0.057
M2	Residence	0.017	0.028	-0.087 ^{a}	0.036
	FA_EDU	0.000	0.033	-0.003	0.042
	MO_EDU	-0.015	0.033	-0.034	0.042
	OBQ_RT	0.117 ^{b}	0.042	-0.034	0.053
	OBQ_PC	-0.023	0.038	0.028	0.049
	OBQ_ICT	0.277 ^{c}	0.034	-0.173 ^{c}	0.044
	FES_COH	-0.159 ^{c}	0.030	0.051	0.038
	FES_CON	-0.185 ^{c}	0.030	0.058	0.038
	FES_EXP	-0.053 ^{a}	0.025	0.030	0.032
M3	Gender	0.013	0.024	0.123 ^{c}	0.030
	FA_EDU	-0.030	0.027	0.007	0.033
	MO_EDU	0.119 ^{a}	0.048	-0.249 ^{c}	0.060
	OBQ_RT	0.129 ^{b}	0.041	-0.044	0.051
	OBQ_PC	-0.038	0.038	0.033	0.047
	OBQ_ICT	0.279 ^{c}	0.034	-0.159 ^{c}	0.042
	FES_COH	-0.169 ^{c}	0.030	0.067	0.036
	FES_CON	-0.191 ^{c}	0.030	0.075 ^{a}	0.037
	FES_EXP	-0.030	0.047	-0.017	0.057
M4	Gender	0.014	0.024	0.122 ^{c}	0.03
	Residence	0.002	0.026	-0.098 ^{b}	0.032
	FA_EDU	-0.002	0.026	-0.018	0.032
	OBQ_RT	0.133 ^{b}	0.041	-0.050	0.051
	OBQ_PC	-0.041	0.038	0.040	0.047
	OBQ_ICT	0.275 ^{c}	0.034	-0.158***	0.043
	FES_COH	-0.176 ^{c}	0.030	0.083 ^{a}	0.036
	FES_CON	-0.197 ^{c}	0.030	0.083 ^{a}	0.037
	FES_EXP	0.067 ^{b}	0.026	-0.210 ^{c}	0.032
M5	Gender	0.014	0.024	0.122 ^{c}	0.030
	Residence	-0.014	0.025	-0.073 ^{a}	0.031
	MO_EDU	0.102 ^{a}	0.045	-0.209 ^{c}	0.056
	OBQ_RT	0.132 ^{b}	0.041	-0.046	0.050
	OBQ_PC	-0.041	0.038	0.037	0.047
	OBQ_ICT	0.278 ^{c}	0.034	-0.165 ^{c}	0.042
	FES_COH	-0.169 ^{c}	0.030	0.070	0.036
	FES_CON	-0.193 ^{c}	0.030	0.075 ^{a}	0.037
	FES_EXP	-0.015	0.044	-0.041	0.054

Notes: I, intercept; S, slope; M1: The Conditional LGCM (integrated all sociodemographic characteristics); M2: The Conditional LGCM (leave gender out); M3: The Conditional LGCM (leave residence out); M4: The Conditional LGCM (leave mo_edu out); M5: The Conditional LGCM (leave fa_edu out); ^{a} $P < .05$; ^{b} $P < .01$; ^{c} $P < .001$.

To assess potential multicollinearity among the independent variables in the structural equation model (SEM), we computed the Variance Inflation Factor (VIF) for each predictor using SPSS 25.0. The results of the multicollinearity analysis are presented in Table S9. All VIF values were below the commonly accepted threshold (e.g., < 5), indicating that multicollinearity was not a concern among the independent variables in this study.

Table S9 Variance Inflation Factor for each predictor variable

Variable	VIF
FES_COH	1.524
FES_CON	1.532
FES_IND	1.093
OBQ_RT	2.967
OBQ_PC	2.515
OBQ_ICT	1.961

Reference

McDonald, K.L., Lochman, J.E., 2012. Predictors and Outcomes Associated with Trajectories of Revenge Goals from Fourth Grade through Seventh Grade. J Abnorm Child Psychol 40, 225–236. https://doi.org/10.1007/s10802-011-9560-0

Nylund, K.L., Asparouhov, T., Muthén, B.O., 2007. Deciding on the Number of Classes in Latent Class Analysis and Growth Mixture Modeling: A Monte Carlo Simulation Study. Structural Equation Modeling: A Multidisciplinary Journal 14, 535–569. https://doi.org/10.1080/10705510701575396

Wu, Y.-Q., Liu, F., Chan, K.Q., Wang, N.-X., Zhao, S., Sun, X., Shen, W., Wang, Z.-J., 2022. Childhood psychological maltreatment and internet gaming addiction in Chinese adolescents: Mediation roles of maladaptive emotion regulation strategies and psychosocial problems. Child Abuse Negl 129, 105669. https://doi.org/10.1016/j.chiabu.2022.105669
